# Editorial

**DOI:** 10.1590/bjpt-rbf.2014.0129

**Published:** 2015

**Authors:** Deborah A. Nawoczenski

**Affiliations:** Guest Co-editor, SPORTS Special Issue Brazilian Journal of Physical Therapy

In August 2016, more than 10,000 athletes representing over 200 countries will converge in Rio de Janeiro for the 'biggest sporting event on the planet'^1^ and the first Olympic Games ever held in South America. In September 2016, another 4000 athletes will be participating in the Paralympics. The world's elite athletes will be competing in 42 events for the Olympics and 22 events for the Paralympics. What an exciting time for Brazil!

This special issue on SPORTS in the Brazilian Journal of Physical Therapy comes at an opportune time for practitioners who work with athletes of all levels of ability and throughout various phases of their rehabilitation recovery. With contributions by an international group of 'elite' practitioners in their field, readers will be sure to find their topic of interest in this special SPORTS issue. These topics range from expert clinical commentaries and critical reviews to presentations of original research related to common sports injuries.

While the World Cup generates an international enthusiasm for soccer, it also informs the viewer about the injuries to soccer players. The article "SPORTS INJURIES PROFILE OF A FIRST DIVISION BRAZILIAN SOCCER TEAM: A DESCRIPTIVE COHORT STUDY" by Reis and colleagues provides information from a descriptive cohort study regarding injury profiles in first division Brazilian soccer players, including the influence of player's age and position on injuries. An appropriate and relevant follow up to this manuscript is presented by Hespanhol Junior and colleagues: "MEASURING SPORTS INJURIES ON THE PITCH: A GUIDE TO USE IN PRACTICE." This paper reviews the basic concepts of injury monitoring systems in sports participation and encourages the implementation of these concepts in practice.

Runners are featured in the manuscript "MALE AND FEMALE RUNNERS DEMONSTRATE DIFFERENT SAGITTAL PLANE MECHANICS AS A FUNCTION OF STATIC HAMSTRING FLEXIBILITY". In this article, Blase Williams and colleagues assess the effect of hamstring length on running mechanics in both male and female runners and discuss the implications for injury. For athletes who have sustained a knee injury, the timing for when it is safe to return to sports can be challenging. In the manuscript "A CONCEPTUAL FRAMEWORK FOR A SPORTS KNEE INJURY PERFORMANCE PROFILE (SKIPP) AND RETURN TO ACTIVITY CRITERIA (RTAC)," Logerstedt and colleagues discuss a comprehensive system that focuses on specific indicators of rehabilitation progression, and present criteria for safe return to sports following knee injury.

Patellar tendinopathy is a common problem in athletes whose sports require jumping. In the article "PHYSICAL THERAPISTS' ROLE IN PREVENTION AND MANAGEMENT OF PATELLAR TENDINOPATHY INJURIES IN YOUTH, COLLEGIATE, AND MIDDLE-AGED INDOOR VOLLEYBALL ATHLETES" Kulig and colleagues discuss intervention strategies that include education, rehabilitation, training and return to sport that are athlete-specific. Patellar tendinopathy is not the only condition linked to sports involving jumping. Achilles tendinopathy is also common and highly problematic in jumping activities. In the manuscript "CLINICAL COMMENTARY OF THE EVOLUTION OF THE TREATMENT FOR CHRONIC PAINFUL MID-PORTION ACHILLES TENDINOPATHY" Alfredson discusses the results of research that evolved and changed practice, including the newest treatment for Achilles tendinopathy.

Given that Golf will be returning to the 2016 Summer Games for the first time in 112 years, Evans and Tuttle's "IMPROVING PERFORMANCE IN GOLF: CURRENT RESEARCH AND IMPLICATIONS FROM A CLINICAL PERSPECTIVE" is a well-timed contribution. Using best evidence from biomechanical and motor control research, the manuscript offers a pragmatic approach to enhancing golf performance.

Core stability is frequently a focus of an athlete's rehabilitation program, yet there is little evidence to support the link between core stability and injury. The article by Silfies and colleagues, "CRITICAL REVIEW OF THE IMPACT OF CORE STABILITY ON UPPER EXTREMITY ATHLETIC INJURY AND PERFORMANCE" provides an in-depth review of the existing science regarding core stability and its association between upper limb injuries and athletic performance. The upper limb is also featured in the article by Cools and colleagues, "PREVENTION OF SHOULDER INJURIES IN OVERHEAD ATHLETES: A SCIENCE BASED APPROACH." The authors discuss the key risk factors that may be used to guide injury prevention and return to sports after shoulder injury.

Finally, an in-depth commentary regarding the importance of study design for injury risk prediction is presented in the manuscript by Hewett and colleagues "MULTI-CENTER TRIAL OF MOTION ANALYSIS FOR INJURY RISK PREDICTION: LESSONS LEARNED FROM PROSPECTIVE LONGITUDINAL LARGE COHORT COMBINED BIOMECHANICAL -EPIDEMIOLOGICAL STUDIES." In their paper, the authors illustrate the research process and emphasize the need for continued, collaborative work in prospective study designs.

**Passion** and **Transformation**: this is the essence of the emblem^2^ chosen for the Olympic and Paralympic Games in Brazil that 'synthesizes its values and guides its action'. The Passion through sports, reflected in the drive and desire for achievement. Transformation in the pride of creating a new reality for progress. This special issue of SPORTS captures the passion of the practitioner in the clinic/research laboratory who desires to optimize health and performance of all athletes, and likewise challenges physical therapists to continue to strive for the transformation of practice.

Parabéns Brasil!

Deborah A. Nawoczenski PT, PhD Guest Co-editor, SPORTS Special Issue
Brazilian Journal of Physical Therapy
